# Indole-3-acetic acid production by *Streptomyces fradiae* NKZ-259 and its formulation to enhance plant growth

**DOI:** 10.1186/s12866-019-1528-1

**Published:** 2019-07-08

**Authors:** Ei Mon Myo, Beibei Ge, Jinjin Ma, Hailan Cui, Binghua Liu, Liming Shi, Mingguo Jiang, Kecheng Zhang

**Affiliations:** 1 State Key Laboratory of Biology of Plant Diseases and Insect Pests, Institute of Plant Protection, Chinese Academy of Agricultural Sciences, Beijing, China; 2 Biotechnology Research Department, Department of Research and Innovation, Ministry of Education, Kyaukse, Myanmar; 3 Guangxi Key Laboratory of Utilization of Microbial and Botanical Resources, School of Marine Sciences and Biotechnology, Guangxi University for Nationalities, Nanning, China

**Keywords:** Indole-3-acetic acid, Optimal conditions, TLC, HPLC, Formulation, Plant growth promotion

## Abstract

**Background:**

Indole-3-acetic acid (IAA) is produced by microorganisms and plants via either tryptophan-dependent or tryptophan-independent pathways. Herein, we investigated the optimisation of IAA production by *Streptomyces fradiae* NKZ-259 and its formulation as a plant growth promoter to improve economic and agricultural development.

**Results:**

The maximum IAA yield achieved using optimal conditions was 82.363 μg/mL in the presence of 2 g/L tryptophan after 6 days of incubation. Thin-layer chromatography (TLC) and high-performance liquid chromatography (HPLC) analysis of putative IAA revealed an RF value of 0.69 and a retention time of 11.842 min, comparable with the IAA standard. Regarding product formulation, kaolin-based powder achieved a suspension rate of 73.74% and a wetting time of 80 s. This carrier exhibited good shelf life stability for NKZ-259, and the cell population did not decrease obviously over 4 months of storage at 4 °C. In vivo analysis of plant growth promotion showed that tomato seedlings treated with kaolin powder containing NKZ-259 cells displayed a significant increase in root and shoot length of 7.97 cm and 32.77 cm, respectively, and an increase in fresh weight and dry weight of 6.72 g and 1.34 g. Compared to controls, plant growth parameters were increased almost it two-fold.

**Conclusion:**

Optimising the culture conditions resulted in an almost four-fold increase in IAA secretion by NKZ-259 cells. The results clearly demonstrate that *S. fradiae* NKZ-259 holds great potential for plant growth promotion and IAA production. Furthermore, kaolin-based powder is an effective carrier for NKZ-259 cells and may be useful for commercial applications.

**Electronic supplementary material:**

The online version of this article (10.1186/s12866-019-1528-1) contains supplementary material, which is available to authorized users.

## Background

Actinomycetes are Gram-positive, plant growth-promoting rhizobacteria that promote plant growth either directly or indirectly [1]. These microbes secrete antibiotics, vitamins and enzymes [2] including indole-3-acetic acid (IAA) [3] and play a key role in the decomposition of organic matter [4] and phosphorus solubilisation [5]. IAA is a common natural auxin phytohormone with an indole ring [6] and a product of L-tryptophan metabolism in microorganisms [7]. Various genera of plant growth-promoting bacteria including actinomycetes and fungi improve plant growth via IAA production through L-tryptophan-dependent mechanisms [6]. Some studies revealed that among IAA-producing actinomycetes, *Streptomyces* sp. is the dominant genus for IAA production [8]. IAA plays a role in a plethora of plant developmental and physiological processes, including embryogenesis, organogenesis, vascular differentiation, root and shoot development, trophic growth, and fruit development [9]. However, the ability of *Streptomyces* cultures to form these bioactive products is not a fixed property, and can be greatly increased or completely lost under different nutrition and cultivation conditions. This is because antibiotic biosynthesis is a specific property of microorganisms that is strongly dependent on the culture conditions. Cell growth and antibiotic production can be improved by manipulating culture nutritional and physical parameters. Thus, media composition plays a vital in terms of efficiency and cost. Carbohydrates and nitrogen play key roles as structural and energy compounds in the cell. Numerous cultivation parameters including pH, carbon source, nitrogen source, and L-tryptophan supplementation can affect bacterial growth and IAA yield. Therefore, designing an appropriate fermentation medium is of critical importance in the production of secondary metabolites [10].

Optimisation of fermentation parameters is imperative for maximising the yield in large-scale microbial production, and parameters that improve outputs must be ascertained [11]. The traditional one-factor-at-a-time (OFAT) approach for optimisation can be time-consuming. Nonetheless, it can estimate optimum levels of medium constituents [12, 13]. Problems that diminish yields can be identified and addressed using statistical tools. For example, a Plackett-Burman experimental design and response surface methodology (RSM) analysis using central composite and Box-Behnken designs has been applied to optimise fermentation parameters [14, 15]. In comparison with conventional optimisation methods in which one variable is varied at a time, RSM has several advantages. It provides a wealth of information and is a more economical approach because a small number of experiments are performed to monitor the effects of interactions between independent variables on the response. Traditional optimisation takes a long time to carry out and leads to an increase in cost due to the large number of experiments needed and the quantities of reagents and materials required [16]. Another disadvantage of experimental optimisation using classical methods that change one factor at a time and fix all other factors is that it does not consider the effects of interactions between different factors under study. Rhizosphere actinomycetes have been studied and developed as commercial products using such methods [17].

Since it is not usually convenient to use freshly prepared inocula in the field, biocontrol/plant growth-promoting (PGP) agents have been developed as powders, solids, and liquid formulations that are more suitable for storage, transportation, and application on a commercial scale [18]. Many PGP rhizobacteria (PGPR) have been formulated in liquids, solids, and powders for use as bioherbicides and biofertilisers. Dry formulations (granules or powders) are generally preferred over liquids due to a longer shelf-life and facile transportation and storage. Furthermore, most granular and powder formulations can also be made into liquids and water-based suspensions as required for drenching, spraying and root-dipping applications [19].

*Streptomyces fradiae* NKZ-259 possesses marked antifungal activity against various plant pathogenic fungi including *Botrytis cinerea*, *Curvularia lunata*, *Alternaria alternata, Colletotrichum gloeosporioides, Rhizoctonia cerealis* and *Ustilaginoidea virens* [20]. The present study focused on optimising the parameters affecting IAA production by *S. fradiae* NKZ-259 and assessing formulation for commercial applications. Various studies have attempted to optimise IAA production by microbes. However, few have applied the response surface methodology (RSM) approach. To our knowledge, this is the first study to apply RSM for the optimisation of IAA production by *Streptomyces*.

## Results

### Optimisation of culture conditions for maximising IAA production using the one-factor-at-a-time (OFAT) method

Under optimal conditions, NKZ-259 cells exhibited a maximum growth rate on the ninth day of incubation, peaking at 0.25 g/10 mL of dry weight. However, on the following day the cell density decreased to 0.02 g/10 mL, and the density continued to decrease until the final sampling time after 2 weeks of incubation. During this time, the pH of the fermentation broth decreased from 6.5 to 6, which was not significant.

OFAT optimisation experiments showed that the highest IAA production under optimal medium and culture conditions was 20.46 μg/mL using Gause’s No.1 medium in the presence of 2 g/L tryptophan, hence this was used in subsequent experiments. Under these conditions, the IAA concentration reached 4.16 μg/mL in ISP-1, 5.75 μg/mL in ISP-2, 0.80 in ISP-3, 8.10 μg/mL in ISP-4, 3.94 μg/mL in Bennet medium, 4.65 μg/mL in MS medium, 5.44 μg/mL in Streptomyces medium and 2.79 μg/mL in Tryptic soy agar. NKZ-259 reached maximal growth rate on the ninth day of incubation, suggesting that secondary metabolite production in this strain was not directly correlated with cell growth.

**Fig. 1:**
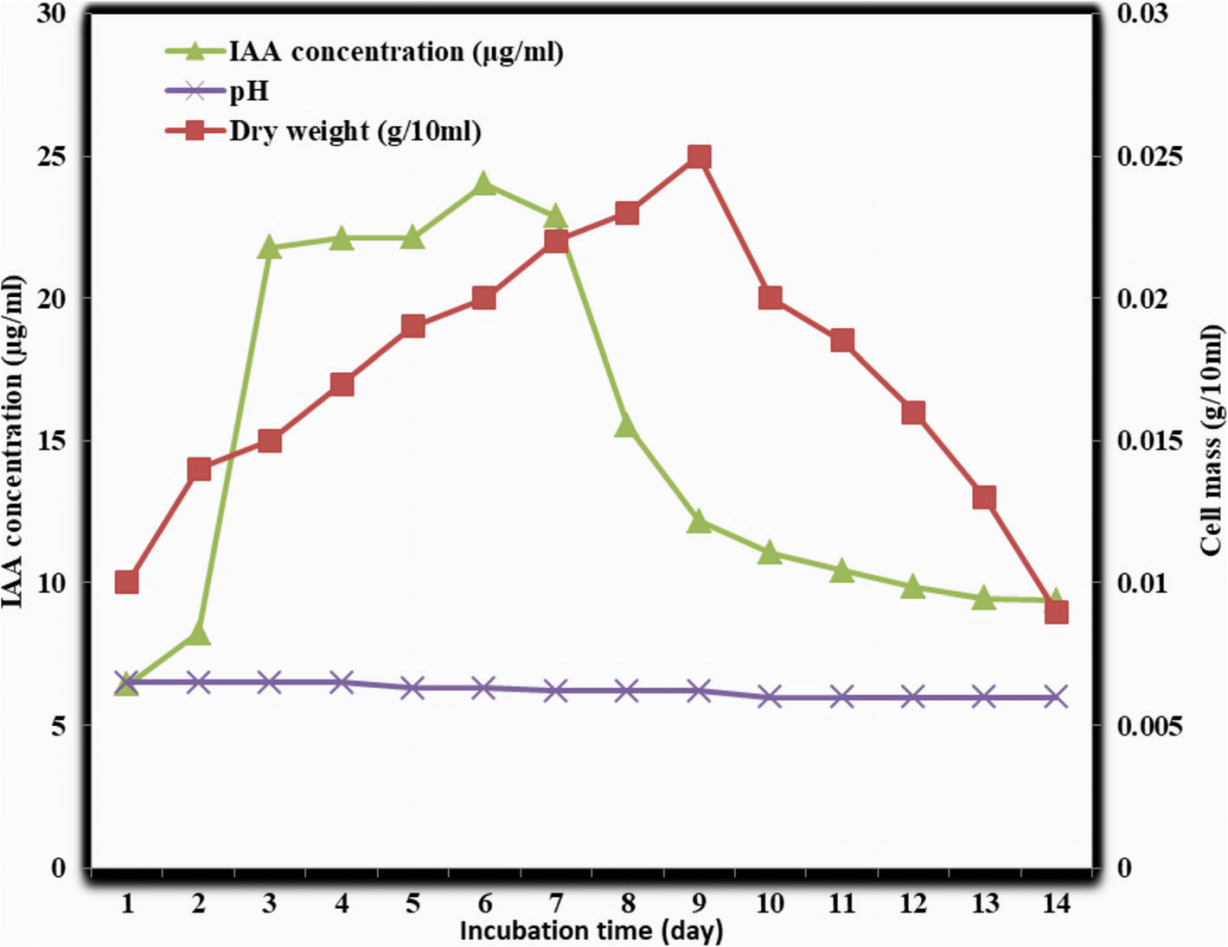
Growth rate and IAA production of NKZ-259 over a period of 2 weeks with respect to pH

The IAA productivity of NKZ-259 was studied for 2 weeks, and day 6 (144 h) was found to be the time of maximum production. On the first day of incubation, only 6.442 μg/mL of IAA was secreted by this strain, but this increased gradually over the following days, peaking at 24.027 μg/mL after 6 days. Changes in cell mass, pH and IAA production for NKZ-259 over the 2 week incubation are illustrated in Fig. 1.

NKZ-259 achieved a maximum yield of 42.345 μg/mL IAA in the presence of starch and KNO_3_, the major substrates in Gause’s No.1 medium. Conversely, IAA production was minimal when sorbitol and casein were added to the medium, reaching only 15.31 μg/ml and 11.771 μg/mL, respectively. This strain produced only a small amount of IAA when carbon and nitrogen sources were omitted from the fermentation medium, demonstrating that these macronutrients had a dramatic effect on PGP hormone production in NKZ-259.

Supplementation of 2 g/L tryptophan in the medium was optimum for IAA production, and NKZ-259 cells secreted up to 56.283 μg/mL IAA. When tryptophan supplementation was increased to 10 g/L, IAA production was only 40.22 μg/mL, and a higher tryptophan concentration had an adverse effect on the IAA production capacity of this potent PGP strain. However, NKZ-259 could produce only 4.876 μg/mL IAA without tryptophan in the medium.

### Optimisation using Plackett-Burman experiments

The Plackett-Burman design (PBD) was applied to examine the fermentation medium factors having the greatest effect on IAA production. Eight potentially important variables (starch, KNO_3_, NaCl, K_2_HPO_4_, MgSO_4_, FeSO_4_, tryptophan and incubation time) were identified by the OFAT method and statistically optimised using the PBD approach. The actual values of each factor and details of the design experiment are shown in (Additional file 1) and (Additional file 2). The results showed that (g/L) X1 (starch)-15, X2 (KNO_3_)-1.5, X3 (NaCl)-0.4, X4 (FeSO_4_)-0.005, X5 (MgSO_4_)-0.4, X6 (K_2_HPO_4_)-0.6, X7 (tryptophan)-3 and X8 (incubation time)-7 were the optimum values for IAA production, which reached 52.058 μg/mL. Only 27.186 μg/mL IAA was secreted when the composition of the medium was (g/L) X1–15, X2–1.5, X3–0.6, X4–0.005, X5–0.6, X6–0.4, X7–1 and X8–5. Statistical analysis of the PBD approach clearly indicated that soluble starch, KNO_3_, NaCl, K_2_HPO_4_, tryptophan and incubation time were the most significant parameters among the eight variables (Table 1). One-way analysis of variance (ANOVA) showed that *p*-values for soluble starch, KNO_3_, NaCl, K_2_HPO_4_, tryptophan and incubation time were significant (0.020, 0.037, 0.004, 0.003, 0.000 and 0.014, respectively), whereas *p*-values for FeSO_4_ and MgSO_4_ were not significant, hence these constituents were omitted in the subsequent RSM analysis. The variance of the actual response was clarified using *R*^2^ values to check the correctness and fitness of the model, and *R*^2^ values could explain 99.38% of the variation in the data (i.e. only 0.62% was not accounted for by the model). The smaller the R-squared value, the less capable the model is for clarifying the variation in dependent variables. The regression equation in uncoded units for this design was as follows:

**Table 1:** Statistical analysis of the Plackett-Burman design for IAA production by *Streptomyces fradiae* NKZ-259

Variable/Term	Coefficient	SE coefficient	t-value	*p*-value
Constant	37.923	0.481	78.77	0.000
X1	2.198	0.481	4.56	0.020^*^
X2	1.731	0.481	3.60	0.037^*^
X3	−4.055	0.481	−8.42	0.004^*^
X4	−1.453	0.481	− 3.02	0.057
X5	0.729	0.481	1.51	0.227
X6	4.142	0.481	8.60	0.003^*^
X7	7.864	0.481	16.33	0.000^*^
X8	−2.479	0.481	−5.15	0.014^*^

R-sq = 99.38% R-sq (adj) = 97.74%

SE, standard error; t, Student’s test; *p*, corresponding level of significance; ^*^, significance at *p* < 0.05

**Fig. 2:**
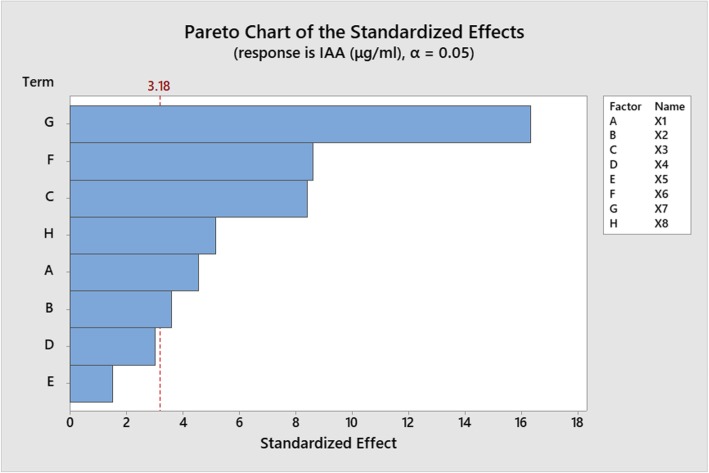
Pareto chart of the standardised effects of different parameters of the plant growth-promoting ability of *Streptomyces fradiae* NKZ-259

IAA (μg/mL) = 23.64 + 0.4395 X1 + 3.462 X2 – 40.55 X3 – 290.6 X4 + 7.29 X5 + 41.43 *X*6 + 7.864 X7 – 2.479 X8.The factors having the greatest effect on IAA production are summarised in the Pareto chart [21] in Fig. 2.

### Optimisation of process variables by response surface methodology (RSM) analysis

The Box-Behnken design was used to further optimise the six factors identified as significant using PBD analysis. The actual values of the process variables are listed in (Additional file 3), and the matrix design and actual response values for IAA productivity are shown in (Additional file 4). In this design, run 40 comprising (g/L) A (starch)-20, B (KNO_3_)-1, C (NaCl)-0.5, D (K_2_HPO_4_)-0.4, E (tryptophan)-2 and F (incubation time)-6 was optimum for IAA production, which reached 82.363 μg/mL. The lowest IAA yield of 22.288 μg/mL was secreted when the composition was (g/L) A-20, B-0.5, C-0.6, D-0.5, E-3, and F-6. Table 2 summarises the results of ANOVA. The very low *p*-value (*p* < 0.0001) suggests that the model fits the experimental data very well, and the Model F-value of 9.26 also indicates high model accuracy. In this case, model terms A, B, C, F, DF, A^2^, B^2^, C^2^, E^2^ and F^2^ were significant, while values greater than 0.1000 were not significant. An F-value for the Lack of Fit of 1.52 demonstrated that it was not significant, suggesting model fitting was good, with only a 34.30% chance that it could be due to noise. A Pred R-squared value of 0.5471 was in accordance with an Adj R-squared value of 0.6858, and the ANOVA equation describing the predicted response was as follows:

$$ \mathrm{Y}\ \left(\mathrm{predicted}\ \mathrm{response}\right)=73.89+5.71\ast \mathrm{A}+5.93\ast \mathrm{B}+4.76\ast \mathrm{C}\hbox{-} 3.39\ast \mathrm{D}\hbox{-} 2.12\ast \mathrm{E}+7.42\ast \mathrm{F}+7.38\ast \mathrm{A}\ast \mathrm{C}\hbox{-} 6.61\ast \mathrm{A}\ast \mathrm{E}\hbox{-} 10.82\ast \mathrm{D}\ast \mathrm{F}\hbox{-} 12.81\ast \mathrm{A}2\hbox{-} 14.71\ast \mathrm{B}2\hbox{-} 12.23\ast \mathrm{C}2\hbox{-} 17.08\ast \mathrm{E}2\kern0.5em 8.67\ast \mathrm{F}2 $$

**Table 2:** Statistical analysis of the Box-Behnken design

Source	Sum of squares	Degree of freedom	Mean square	F-value	*p*-value (prob >F)
Model	14,220.13	14	1015.72	9.26	< 0.0001
A-Starch	781.77	1	781.77	7.13	0.0110
B-KNO_3_	842.58	1	842.58	7.69	0.0085
C-NaCl	542.72	1	542.72	4.95	0.0319
D-K_2_HPO_4_	276.29	1	276.29	2.52	0.1205
E-Tryptophan	108.14	1	108.14	0.99	0.3268
F-Incubation time	1320.17	1	1320.17	12.04	0.0013
AC	435.48	1	435.48	3.97	0.0533
AE	349.03	1	349.03	3.18	0.0822
DF	936.62	1	936.62	8.54	0.0057
A^2^	1836.64	1	1836.64	16.75	0.0002
B^2^	2273.38	1	2273.38	20.74	< 0.0001
C^2^	1569.56	1	1569.56	14.32	0.0005
E^2^	3062.90	1	3062.90	27.94	< 0.0001
F^2^	789.01	1	789.01	7.20	0.0107
Residual	4275.65	39	109.63		
Lack of Fit	3898.03	34	114.65	1.52	0.3430
Pure Error	377.63	5	75.53		

*R*^2^ = 0.7688; Adj *R*^2^ = 0.6858; Pred R-squared = 0.5471

**Fig. 3:**
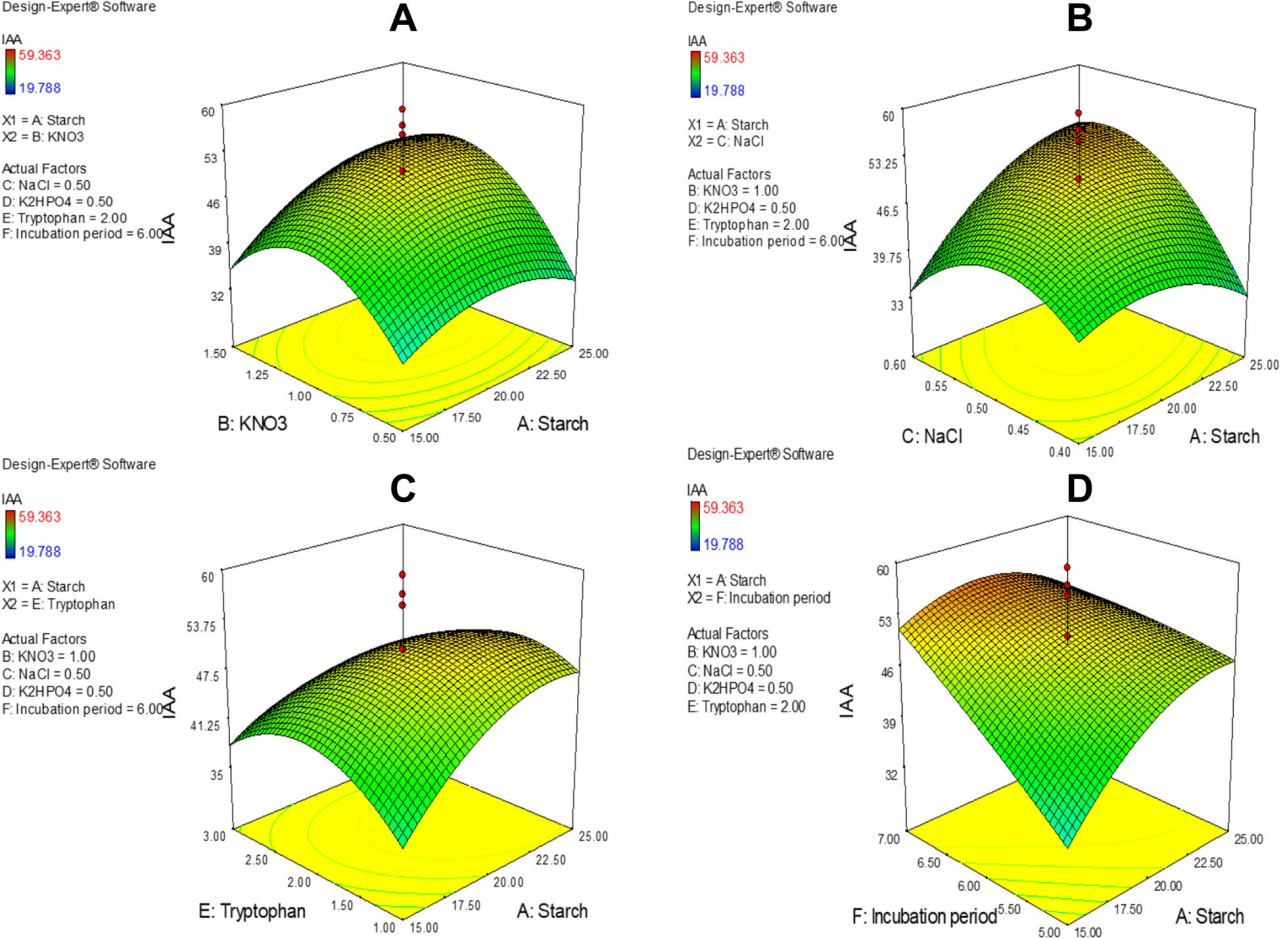
Response surface plots of the effects of various factors on IAA production by NKZ-259. **a** Interaction of A and B, **b** Interaction of A and C, **c** Interaction of A and E, **d** Interaction of A and F

Relationships between significant variables and optimal values for each factor affecting IAA production were assessed using response surface plots [22]. Using the approach, the effects of the two components are considered while those of other variables are kept at zero. Figure 3 shows the relationships between each pair of variables and maximum IAA production by NKZ-259.

### IAA confirmation by thin-layer chromatography (TLC) and high-performance liquid chromatography (HPLC)

**Fig. 4:**
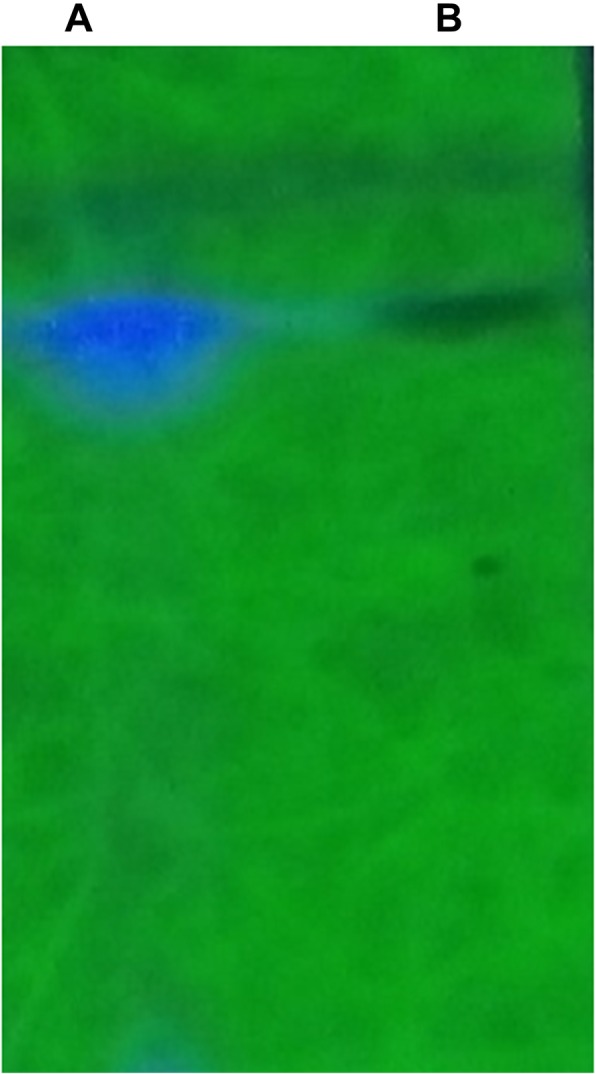
Confirmation of IAA production by NKZ-259 using thin-layer chromatography (TLC). **a** extracted IAA; **b** standard IAA

**Fig. 5:**
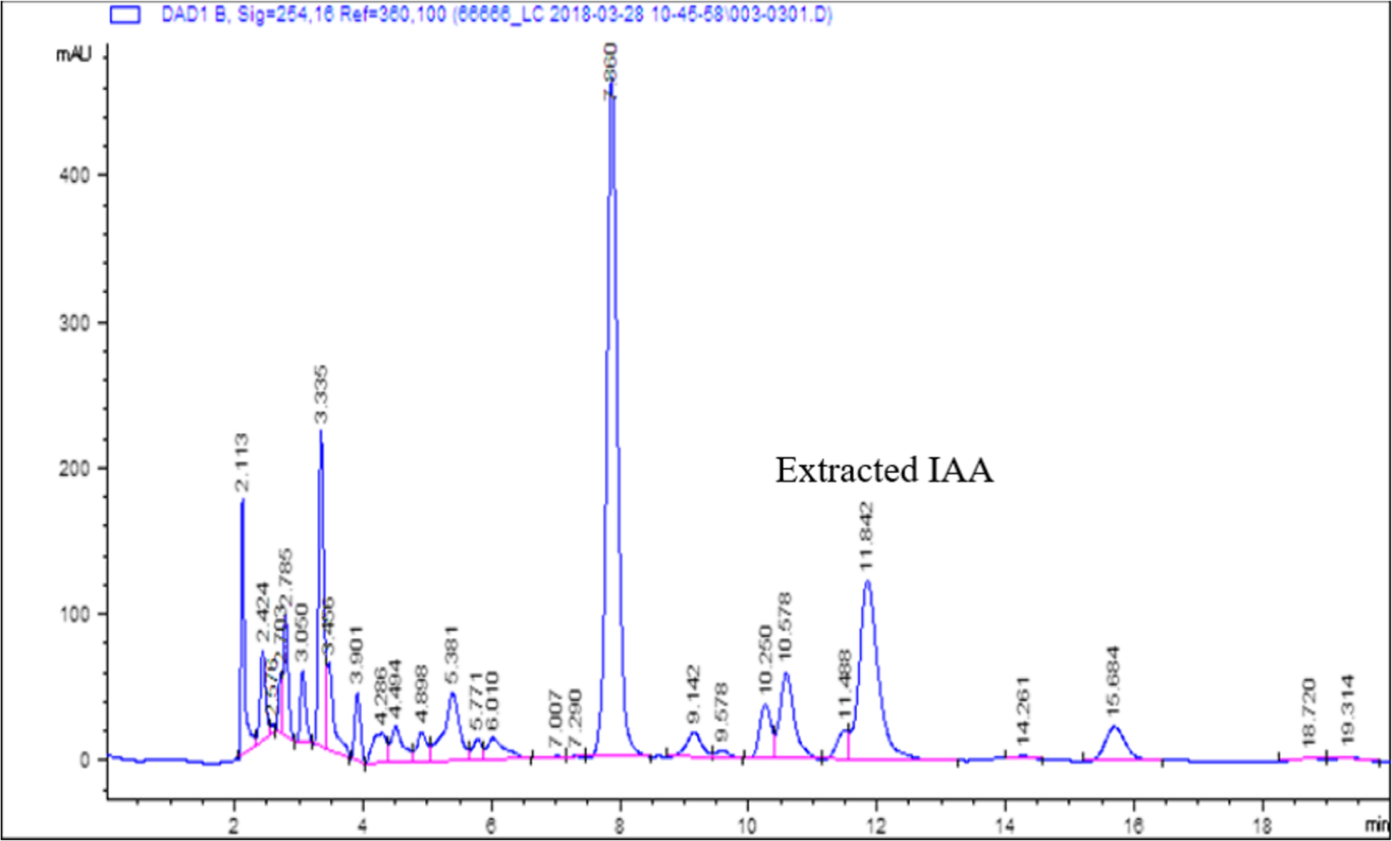
Analysis of IAA production by NKZ-259 using high-performance liquid chromatography (HPLC)

As shown in Fig. 4, spots separated on TLC plates were developed and observed under UV light at 245 nm. The results revealed that a standard IAA sample and putative IAA samples extracted from NKZ-259 cells displayed the same retention factor (RF) value of 0.69. Similarly, the HPLC elution profile of an authentic IAA standard exhibited a major peak at a retention time of 11.852 min, while IAA isolated from NKZ-259 appeared as a sharp peak at a similar retention time of 11.842 min (Fig. 5).

### Germination rate and plant growth promotion by NKZ-259 under greenhouse conditions

In germination assessment, all treatments exhibited 100% germination rates on tomato seeds (Table 3). The effects of NKZ-259 on plant growth promotion, including root development, were assessed. Root elongation assays and pot experiments were performed using tomato seeds inoculated with the PGP NKZ-259 strain. Water, medium, cell-free filtrate and culture treatments were significantly different from each other. In plant growth promotion assays, NKZ-259 cultures significantly enhanced plant growth by increasing the root length, shoot length, fresh weight and dry weight of all plants. After 5 weeks of growth, NKZ-259 cells promoted root length to 8.08 cm, shoot length to 24.20 cm, fresh weight to 2.63 g and dry weight to 0.25 g. In filtrate-treated plants, root and shoot length were enhanced by 7.50 cm and 23.13 cm, respectively, while fresh weight and dry weight were increased to 2.56 g and 0.24 g, respectively. These plant growth parameters were not significantly different from the other two treatments. Table 4 summarises the plant growth-promoting effects of this potent strain on tomato plants.

**Table 3:** Germination tests on tomato plants subjected to representative treatments

Treatment	Germination (%)	Root length (cm/seedling)	Shoot length (cm/seedling)
Distilled water	100 a	4.88 ± 0.0265 b	3.25 ± 0.0500 c
Gause’s medium	100 a	4.92 ± 0.0361 b	4.52 ± 0.0436 b
NKZ-259 cells	100 a	5.48 ± 0.0764 a	5.16 ± 0.0321 a
NKZ-259 filtrate	100 a	5.38 ± 0.0755 a	4.59 ± 0.0451 b

Note: Means with the same letter are not significantly different from each other according to least significant difference (LSD) tests at *p* < 0.05 level. Data were recorded and analysed after 1 week

**Table 4:** Greenhouse analysis of plant growth promotion by NKZ-259 on tomato plants after 5 weeks

Treatment	Root length (cm/plant)	Shoot length (cm/plant)	Fresh weight (g/plant)	Dry weight (g/plant)
Water	5.34 ± 1.86 b	19.53 ± 3.22 b	1.46 ± 0.61 b	0.12 ± 0.06 b
Medium	5.98 ± 1.74 b	20.73 ± 2.05 b	1.83 ± 0.42 b	0.18 ± 0.06ab
Filtrate	7.50 ± 1.06 a	23.13 ± 1.56 a	2.56 ± 0.32 a	0.24 ± 0.05 a
Culture	8.08 ± 1.12 a	24.20 ± 2.32 a	2.63 ± 0.66 a	0.25 ± 0.08 a

Note: Means with different letters are significantly different from each other as determined by least significant difference (LSD) tests at *p* < 0.05

### In vivo analysis of plant growth promotion by the formulated products

**Fig. 6:**
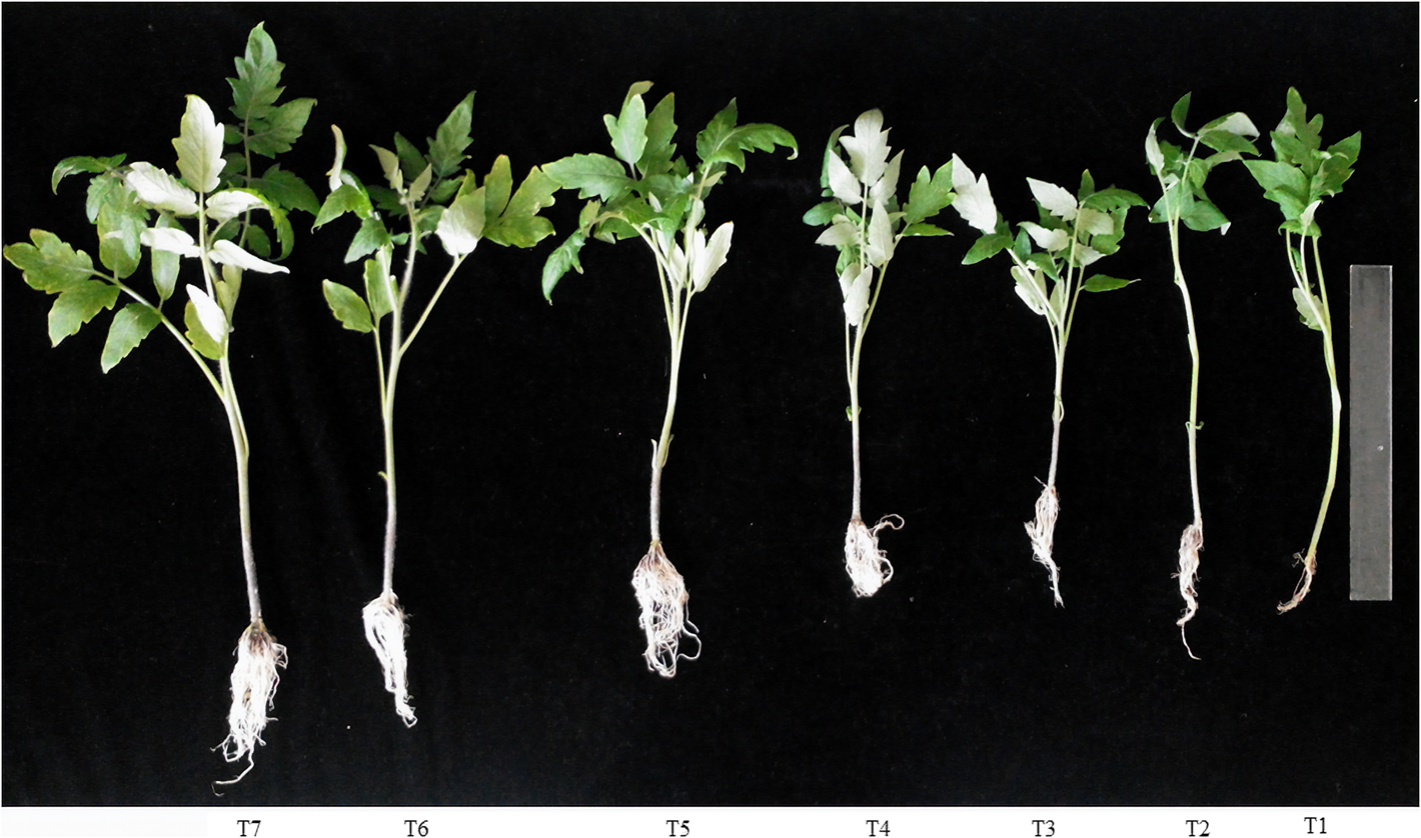
Effect of different treatments on the growth of tomato plants. T1 = water, T2 = kaolin powder without bacterium, T3 = talc powder without bacterium, T4 = molasses and humic acid without bacterium, T5 = talc powder with bacterium, T6 = molasses and humic acid with bacterium, T7 = kaolin powder with bacterium

The effects of each treatment on plant growth were determined at 35 days after planting. Tomato plants treated with each product indicated better PGP effects than control treatments. Among the different formulations, tomato plants treated with NKZ-259 formulated with kaolin-based powder displayed increased shoot length (32.77 cm), root length (7.97 cm), fresh weight (6.72 g) and dry weight (1.34 g) (Fig. 6 and Table 5). This was the only parameter that showed a statistically significant correlation with plant growth promotion. Among the different treatments, minimum plant growth was mediated by water alone.

**Table 5:** In vivo examination of the plant growth promoting effects of NKZ-259 in different formulations

Treatment	Root length (cm)	Shoot length (cm)	Fresh weight (g)	Dry weight (g)
T1	4.18 ± 1.12 d	27.46 ± 2.39 bc	3.87 ± 1.20 b	0.48 ± 0.33 bc
T2	5.33 ± 0.84 cd	24.13 ± 2.13 de	2.57 ± 0.54 b	0.39 ± 0.26 c
T3	5.40 ± 1.33 cd	21.77 ± 2.52 e	2.61 ± 0.71 b	0.38 ± 0.21 c
T4	5.92 ± 5.92 bc	26.00 ± 1.7 cd	3.28 ± 1.19 b	0.32 ± 0.15 c
T5	7.13 ± 1.54 ab	30.80 ± 3.46 ab	6.39 ± 2.45 a	0.97 ± 0.64 ab
T6	6.92 ± 0.95 ab	30.33 ± 2.99 ab	6.65 ± 1.93 a	1.24 ± 0.50 a
T7	7.97 ± 1.26 a	32.77 ± 3.70 a	6.72 ± 2.14 a	1.34 ± 0.68 a

Note: T1 = water, T2 = kaolin powder without bacterium, T3 = talc powder without bacterium, T4 = molasses and humic acid without bacterium, T5 = talc powder with bacterium, T6 = molasses and humic acid with bacterium, T7 = kaolin powder with bacterium. Means with different letters are significantly different from each other as determined by least significant difference (LSD) tests at *p* < 0.05. These characteristics were measured and calculated after 5 weeks of plant growth

### Suspension rate and wetting time of carriers

In our current experiments, the suspension rate of kaolin-based powder was better than that of talc powder, which also displayed a longer wetting time, resulting in very slow suspension in water. By contrast, kaolin powder achieved both a higher suspension rate and better wettability. Table 6 summarises pH, suspension rate and wettability for each representative powder.

**Table 6:** Evaluation of the suspension rate and wetting time of two wettable powders according to national standards

No.	pH	Suspension rate (%)	Wetting time (second)
National standard	–	≥60	≤120
Kaolin powder	8.10 ± 0.36 a	73.74 ± 6.00 a	80.0 ± 6.32 a
Talc powder	8.15 ± 0.36 a	50.55 ± 6.12 b	97.2 ± 14.79 a

### Shelf life analysis

The density of NKZ-259 cells was 5.6 × 10^6^ colony-forming units (CFU)/ml in each formulated product, and the cell count in each carrier was determined at 1-month intervals over 3 months by the serial dilution plate count method. After 1 month of storage at 4 °C, the population of cells in kaolin powder was stable, and the population was almost stable after storage at room temperature. However, the population of NKZ-259 cells in talc and liquid carriers was slightly decreased during storage at both 4 °C and room temperature. After 2 months of storage, the population of cells had declined gradually, but the decrease was slight and not significant in kaolin powder. After 2 months of storage, although the cell populations in kaolin powder kept in a refrigerator or at room temperature were nearly stable at 5.2 × 10^6^ CFU/mL and 2.3 × 10^6^ CFU/mL, respectively, the cell density in talc-based powder kept at 4 °C or room temperature was reduced to 6.5 × 10^4^ CFU/mL and 5.5 × 10^4^ CFU/mL, respectively, compared with 1.0 × 10^4^ CFU/mL in the liquid carrier. The cell density in kaolin powder for both storage conditions remained constant up to 3 months of storage, whereas cells in talc powder decreased slightly to 5.8 × 10^4^ CFU/mL and 4.8 × 10^4^ CFU/mL at 4 °C and room temperature, respectively. However, the NKZ-259 population in the liquid carrier decreased to 1.0 × 10^3^ CFU/mL at both storage temperatures. At the final sampling time (4 months of storage), the number of NKZ-259 cells in kaolin powder had decreased slightly but not significantly. At this time point, the population in talc-based powder had declined to 3.9 × 10^4^ CFU/mL and 2.0 × 10^4^ CFU/mL at 4 °C and room temperature, respectively. Additionally, the number of cells in the liquid carrier had decreased significantly to ~ 0.5 × 10^3^ CFU/mL at 4 °C and ~ 0.2 × 10^3^ CFU/mL at room temperature. Powder formulations kept at 4 °C displayed a longer shelf life than samples stored at room temperature. Among the three formulations, kaolin-based powder was the most suitable carrier for NKZ-259 stability; even after 4 months, the cell population at 4 °C was not obviously decreased (Table 7).

**Table 7:** NKZ-259 cell viability rate in different carriers (original cell count in each carrier = 5.6 × 10^6^ CFU/mL)

Carriers	Cell population (CFU/mL)			
	1 month	2 months	3 months	4 months
Kaolin (4 °C)	5.4 ± 0.15 × 10^6^ a	5.2 ± 0.15 × 10^6^ a	5.2 ± 0.15 × 10^6^ a	5.2 ± 0.15 × 10^6^ a
Kaolin (RT)	5.3 ± 0.14 × 10^6^ a	2.4 ± 0.15 × 10^6^ b	2.3 ± 0.2 × 10^6^ b	1.7 ± 0.2 × 10^6^ b
Talc (4 °C)	1.4 ± 1.15 × 10^5^ b	6.5 ± 0.20 × 10^4^ c	5.1 ± 0.3 × 10^4^ c	3.6 ± 0.25 × 10^4^ c
Talc (RT)	1.0 ± 0.9 × 10^5^ b	5.5 ± 0.25 × 10^4^ c	5.0 ± 0.4 × 10^4^ c	2.1 ± 0.2 × 10^4^ c
Liquid (4 °C)	1.3 ± 0.15 × 10^5^ b	1.1 ± 0.11 × 10^4^ c	1.2 ± 0.15 × 10^3^ c	0.6 ± 0.15 × 10^3^ c
Liquid (RT)	1.0 ± 0.9 × 10^5^ b	1.1 ± 0.11 × 10^4^ c	1.2 ± 0.2 × 10^3^ c	0.4 ± 0.15 × 10^3^ c

Note: 4 °C = storage at 4 °C in a refrigerator; RT = room temperature storage (27 °C)

## Discussion

The statistical optimisation method has been applied to IAA production, but statistical optimisation of medium components for IAA production by *Streptomyces fradiae* based on statistical design is yet to be reported. *Streptomyces* species typically require organic carbon and inorganic nitrogen sources plus mineral salts for growth as well as secondary metabolite production [23]. The IAA-producing activity of PGPR varies among species and is greatly influenced by culture conditions, growth stage and substrate ability [24]. Optimisation of the fermentation medium is imperative for maximising the ability of microbes to produce secondary metabolites on an industrial scale. Although carbon nutrients are essential, nitrogen sources and other micronutrients should not be neglected when optimising production [25].

The results of OFAT experiments showed that in addition to nutrients in the fermentation medium, tryptophan and incubation time played an important role in IAA production by NKZ-259; IAA production was improved from 4.876 μg/mL IAA in the absence of tryptophan to 56.28 μg/ml in its presence, strongly indicating that tryptophan is a precursor for IAA production. Furthermore, NKZ-259 appears to secrete IAA via a tryptophan-dependent biosynthetic pathway. This finding is consistent with the knowledge that actinomycetes possess the ability to produce the auxin phytohormone IAA in the presence of a suitable precursor such as L-tryptophan [26]. However, other pathways may be included in this mechanism because some bacteria possess more than one pathway [27].

Following OFAT optimisation, we optimised the fermentation medium, tryptophan supplementation, and incubation time using the Plackett-Burman design (PBD) and Box-Behnken design. NKZ-259 produced only 20.46 μg/mL IAA in the original fermentation medium in the presence of tryptophan, but the yield was increased to 82.363 μg/mL after this round of optimisation, equating to a four-fold increase in IAA produced. These findings are consistent with previous reports demonstrating the advantages of the RSM approach [28].

TLC analysis of IAA standards and extracted samples revealed identical Rf values, consistent with previous studies [29]. HPLC is a more reliable and powerful method for identifying and analysing auxins than mass spectrometry [30]. Thus, we characterised IAA extracted from NKZ-259 by HPLC. The retention times of sample peaks were comparable to those of authentic IAA standards, confirming that strain NKZ-259 produced IAA and was indeed a PGPR member.

In vivo plant growth promotion assays showed that plants treated with kaolin powder containing NKZ-259 exhibited the highest root and shoot lengths, as well as fresh and dry weights. Although plants treated with T2 (kaolin powder without bacteria), T3 (talc powder without bacteria), T4 (molasses and humic acid without bacteria) displayed increased root elongation superior to that of plants treated with T1 (water), other characteristics were inferior to those of T1-treated plants. Plants treated with carriers containing NKZ-259 displayed significantly increased growth, and those treated only with kaolin and talc-based powders did not exhibit significant plant growth promotion. Thus, the NKZ-259 strain early promoted plant growth, which may be due to metabolites produced by NKZ-259 other than IAA. However, at nearly every developmental stage (embryonic and postembryonic) and in every growth process (formation of lateral organs and growth of leaves), plants were affected by IAA directly or indirectly via secondary induced signalling molecules [31]. IAA compounds produced by NKA-259 played an essential role in plant growth promotion because the main function of auxins is to stimulate root elongation, and this improvement was obvious in NKZ-259 treated plants.

The national standard for the suspension rate of a wettable powder is ≥60% and the standard wetting time is ≤120 s [32]. Our results show that the kaolin-based product meets these criteria because the suspension rate was 73.74% and the wettability was 80 s. Furthermore, shelf life analysis showed that the longevity (stability) of NKZ-259 was highest in kaolin powder. This PGP strain was 100% stable after storage for 1 month, and almost completely stable up to 2 months, and stability was only slightly decreased after 3 months of storage. At the final sampling time (4 months), there was no significant decrease in the NKZ-259 cell population in kaolin powder, whereas a significant decline was observed with the other two carriers. The stability and viability of bacteria in formulations during storage may be influenced by the nutritional supplements added [33]. Our results suggest that the type of carrier in the formulation may affect cell viability. Kaolin-based powder appears to be the most suitable carrier for NKZ-259 formulations intended for agricultural use.

## Conclusions

In this work, we optimised IAA production by NKZ-259 using an RSM approach to explore formulations best suited for commercial use in agriculture. A suitable medium for improved IAA production was successfully established, and IAA production was elevated by optimising the culture conditions using a statistical design method. In greenhouse assays, both NKZ-259 and the formulated carrier containing NKZ-259 cells could promote growth of tomato plants. The PGP bacterium was very stable in the kaolin-based carrier, which may be of significance for agricultural applications. Finally, the results provide strong evidence that *Streptomyces fradiae* NKZ-259 is a promising and effective PGPR inoculant for plant growth promotion that may enrich soil fertility and enhance crop yields.

## Methods

### Collection of *Streptomyces fradiae* NKZ-259

NKZ-259 cells were kindly provided by the Functional Genome and Gene Safety Laboratory, Institute of Plant Protection, Chinese Academy of Agricultural Sciences, Beijing, China. This PGP bacterium was collected from soil samples obtained from Qilian Mountain, Qinghai, China, from irrigated but not agricultural land. Sequences have been deposited at GenBank under Accession number CP032266. NKZ-259 was sub-cultured on mannitol soya flour agar (MS) medium containing 20 g/L agar, 20 g/L mannitol, 20 g/L soya flour, and 10 mM CaCl2 1.109 g/L [34] and stored at 4 °C for future use.

### Optimising conditions for IAA production by NKZ-259

For optimum culture conditions, growth medium, IAA production medium, incubation time, carbon and nitrogen sources, and tryptophan concentration were optimised by the OFAT method [35, 36]. The growth rate of cells was investigated using the conventional oven method [37] to measure dry weight and biomass [38]. Briefly, NKZ-259 cells were grown in various media an after 4 days of incubation they were filtered through a No. 1 Whatman filter paper, dried at 40 °C for 1 h, and the dry weight of the biomass was measured. Additionally, pH changes in the culture medium were noted every 24 h for 2 weeks. In all experiments, the inoculum size was 5.6 × 10^6^ CFU/mL of NKZ-259 cells.

IAA production by NKZ-259 was determined using nine different media types; ISP-1, ISP-2, ISP-4, glucose-yeast extract-malt extract (GYM) [39], Bennet’s medium, Gause’s No.1 medium [40], MS medium, *Streptomyces* medium [41] and tryptic soy medium [42]. NKZ-259 cells were inoculated in the above media at 28 °C on a rotary shaker with shaking at 200 rpm. After 4 days of incubation, cells were centrifuged at 12,000 g for 10 min and the supernatant was collected. The IAA concentration was measured by colorimetric assay [43] using Salkowski reagent. The pink colour of individual assays was measured using an Infinite 200 Pro NanoQuant UV-vis spectrophotometer (Tecan, Switzerland) at 530 nm. The IAA concentration was also investigated by colorimetric assay using Salkowski reagent. Before optimising, NKZ-259 cells produced 20.46 μg/mL of IAA in Gause’s No.1 medium (pH 6.5) containing 20 g/L soluble starch, 0.5 g/L NaCl, 0.01 g/L, FeSO_4_·7H_2_O, 0.5 g/L K_2_HPO_4_, 1 g/L KNO_3_ and 0.5 g/L MgSO_4_·7H_2_O. To determine the optimum incubation time, IAA production by NKZ-259 was examined at 24 h intervals for 2 weeks.

For carbon and nitrogen nutrient optimisation, sucrose, glucose, lactose, maltose, mannitol, sorbitol, glycerol and starch were tested as carbon sources, and beef extract, yeast extract, malt extract, soybean flour, casein, tryptone, soy peptone, KNO_3_ and (NH_4_)_2_SO_4_ were tested as nitrogen sources. During optimisation experiments, all other minor nutrients in the medium were kept constant.

Different tryptophan concentrations (0, 1, 2, 3, 4, 5, 6, 7, 8, 9 and 10 g/L) were tested for IAA production using a previously described method [44] with some modifications. All experiments were performed in triplicate three times to obtain average values.

### Optimising the fermentation medium using the Plackett-Burman design (PBD) approach

After optimising single factors, the Plackett-Burman experimental design was applied to screen variables among the six nutrient factors (soluble starch, KNO_3_, NaCl, K_2_HPO_4_, MgSO_4_ and FeSO_4_) [45].

Optimisation using the OFAT method suggested that incubation time and tryptophan may be important factors affecting IAA production by NKZ-259, and these parameters were also optimised by the PBD method. Parameters were screened in 12 experiments and the IAA concentration was used as the response value. All variables were evaluated at two widely-spaced intervals, expressed as negative values (low level, − 1) and positive values (high level, + 1). The Minitab 17.0 statistical software package (State College, PA 2010) was used for Plackett-Burman experimental design and analysis of the results.

### Response surface methodology using the box-Behnken design

After optimising process variables by PBD, the most significant six variables (soluble starch, KNO_3_, NaCl, K_2_HPO_4_, tryptophan and incubation period) were further subjected to RSM analysis using the Box-Behnken design [46] with the Design-Expert 9.0 statistical software package (Minneapolis, MN, USA).

A set of 54 experiments was generated and each factor was assessed at three levels (+ 1, 0 and − 1) where 0 is a central coded value, + 1 is a high value, and − 1 is a low value. These experiments were carried out in triplicate and the average IAA concentration was calculated and taken as the actual response. Predicted response values were also calculated using the regression equation.

### Extraction, purification and determination of IAA

NKZ-259 cells were inoculated using the optimum culture conditions, and IAA was separated and purified using a previously reported method [47]. Briefly, NKZ-259 cells were incubated in optimised Gause’s No.1 medium at 28 °C with shaking at 200 rpm for 6 days. The fermentation broth was filtered using a Whatman No.1 filter paper and the culture filtrate was adjusted to pH 9 with 1 M NaOH to keep IAA ionised and more polar. The filtrate was partitioned against 100% ethyl acetate and the upper organic phase was recovered. The pH was lowered to 3 with concentrated acetic acid to preserve IAA in the solvent, and the sample was evaporated to dryness. The dried compound was dissolved in 3 ml of analytical grade methanol and used for TLC and HPLC analyses. The IAA standard was prepared at a concentration of 500 ppm. Standard IAA and extraction samples were spotted on aluminium-backed silica gel G plates and TLC was performed with a mobile phase of butanone: ethyl acetate: ethanol: water (3:5:1:1, v/v/v/v) [48]. TLC plates were dried and spots were observed under UV light at 256 nm.

The IAA standard and extracted samples were analysed by chromatography on an Agilent 1100 modular HPLC system (Agilent Technologies, Germany). The column temperature was kept at 25 °C. Methanol and 1% acetic acid (60:40 v/v) was used as the mobile phase at a flow rate of 1 mL/min [49] and the injection volume was 20 μL. Detection was monitored at 254 nm and 280 nm, and data were evaluated using Chem Station Plus.

### Assessment of tomato (*Solanum lycopersicum* L.) germination rate

Tomato seeds (Qiangfeng 70 White Fruit) were collected from the government compound of Shuanggang town, Jinnan District, Tianjin, China. This is a new variety selected from a single Qingfeng Tomato plant by the Shuanggang Agricultural Technologies Station of Jinnan District, Tianjin, China. In this assay, tomato seeds were firstly disinfected by soaking in 80% ethanol for 3–5 min, followed by 0.2% sodium hypochlorite for 3 min, and washed thoroughly with sterilised distilled water three times. Samples were then dried under laminar flow for the next step. Sterilised seeds were soaked in four treatments; (distilled water, Gause’s No.1 medium only, NKZ-259 cell suspensions, and the NKZ-259 filtrate) for 2 h. Ten seeds from each treatment (with three replicates) were then placed in a Petri dish containing sterile wet tissue paper and kept under semi-dark conditions at 26 ± 1 °C for 1 week, after which time germination rates and shoot and root lengths were measured.

### Evaluation of the PGP potential of NKZ-259 on tomato plants under greenhouse conditions

Firstly, soil, compost and vermiculite were mixed at a ratio of 3:3:1 (pH 6.5) and sterilised by autoclaving at 121 °C for 30 min [50]. Tomato seeds were also sterilised using the method described above. The four treatments described above for germination tests were also performed in this assay. For sample preparation, NKZ-259 cells were incubated in Gause’s No.1 medium for 6 days to reach a cell density of 5.6 × 10^6^ CFU/mL. For cell suspension treatments, cells were used without any filtration. For other treatments, the fermentation culture was filtered through a Whatman filter paper and the filtrate was used. For medium treatments, sterilized Gause’s No.1 medium was used after autoclaving. After preparing each representative treatment, sterilized seeds were soaked in each treatment for ~ 2 h on a rotary shaker at 200 rpm and 28 °C. After treatment, two seeds were transferred to each 20 cm diameter plastic pot filled with 500 g of a sterilised soil mixture, and 2 mL of each bacterial treatment (5.6 × 10^6^ CFU/mL) was sprayed on each plant once a week. Plants were watered with sterilised distilled water every other day. The experiment was carried out in a greenhouse with a light intensity of 2000 lx for 16 h daily at 25 ± 2 °C. A completely randomised design with ten replicates for each treatment was applied. After 5 weeks, the root length, shoot length, plant height, fresh weight and dry weight of treated plants were measured for each treatment.

### Formulation of NKZ-259 as a plant growth promoter

A previous method [51] was modified and applied for wettable talcum powder preparation. The kaolin-based formulation was prepared by mixing 0.3% sodium alginate (dispersant), 10 g of starch, 100 g of kaolin and 0.2 M CaCO_3 (_pH adjustment) and sterilising by autoclaving at 121 °C for 30 min. After sterilising, 250 ml of cell suspension (5.4 × 10^6^ CFU/mL) was added and the volume was made up to 500 ml with sterilised water. The wettable powder was then dried at 37 °C overnight.

For liquid formulation, molasses and humic acid were firstly screened for both cell growth and IAA production. Optimised concentrations of 1% humic acid 30% molasses were subsequently used as carriers. Each product was stored at 4 °C and room temperature in the dark, and the shelf life of NKZ-259 in each product was investigated monthly.

### Analysis of plant growth promotion by the formulated products under greenhouse conditions

Seven treatments (kaolin powder with bacterial strain, talc powder with bacteria, liquid carrier with bacteria, kaolin powder without bacteria, talc powder without bacteria, liquid carrier without bacteria, and sterile distilled water) were evaluated for PGP activity in a completely randomised design with 10 replicates. Tomato seeds were sterilised using the method described above. Two seeds were transferred to each 20 cm diameter pot containing 500 g of sterilised soil mixture, 2 g of each representative sample was completely dissolved in 10 ml of sterilised distilled water, and 2 mL of each treatment was used to treat plants at weekly intervals. The experiment was carried out in a greenhouse with a light intensity of 2000 lx and a 16 h light / 8 h dark photoperiod with a relative humidity of 75% at 25 ± 2 °C. After 5 weeks, various growth characteristics of treated plants including root length, shoot length, plant height, fresh weight and dry weight were measured.

### Determination of the suspension rate and wetting time of each carrier

In accordance with the national wettable powder suspension rate determination standard GB/T 14825–93, 1 g of each sample was accurately weighed and added to a bottle containing 50 mL standard hard water, vigorously vibrated and shaken at 120 rpm for 2 min until uniformly distributed. Samples were then placed in a 30 °C water bath, and after a 13 min incubation, standard hard water was added to a volume of 250 mL. The bottle was inverted ~ 30 times per min, the plug was removed, and the bottle was stored vertically in a stable temperature water bath for 30 min. Next, 90% of the upper suspension was transferred into a beaker and the remaining 10% was completely removed, dried in an oven at 54 °C, and the suspension rate (X) was calculated according to the formula [32]:

$$ \mathrm{X}=\left({\mathrm{m}}_1\hbox{-} {\mathrm{m}}_2\right)/\left({\mathrm{m}}_1\times 10/9\right)\times 100\%. $$

Where m_l_ is the quantity of the sample (g), and m_2_ is the quantity of the 10% residue (g).

The wetting time of each powder was determined as reported by the Chinese pesticide wettable powder wetting time determination standard GB/T 5451–01 [52]. Briefly, a 250 mL beaker containing 100 mL standard hard water was placed in the water bath at 25 °C. At that time, the water bath surface and the liquid surface of the sample was the same level. Then, the weighted 1 g sample was immediately placed into the breaker. The liquid surface was stationary, with no movement, and the wetting time was measured immediately after removing the sample. The experiment was repeated three times and the average value for the wetting time was recorded.

### Shelf life analysis of NKZ-259 in each carrier

The stability of the PGP strain in the carrier is an important factor for commercial production and application. In this case, the viable NKZ-259 cell count in each product was measured after storage at 4 °C and room temperature using the serial dilution and plate count method at regular intervals, usually once a month.

### Analysis of variance (ANOVA)

A completely randomised design was used throughout the experiment, and data analysis was evaluated by One-way ANOVA followed by least significant difference (LSD) tests at *p* < 0.05 using the Minitab 17.0 statistical software package (State College, PA 2010).

## Additional files

Additional file 1: **Table S1.** Actual values of process variables in 1000 mL of fermentation medium. (DOCX 13 kb)

Additional file 2: **Table S2.** The Plackett-Burman experimental design. (DOC 37 kb)

Additional file 3: **Table S3.** Actual values of process variables in 1000 mL of fermentation medium. (DOC 29 kb)

Additional file 4: **Table S4.** The Box-Behnken experimental design. (DOC 89 kb)

## Data Availability

All data generated or analysed during this study are included in this published article (and its supplementary information files).
