# Supplementary material for: Indole-3-acetic acid production by Streptomyces fradiae NKZ-259 and its formulation to enhance plant growth
Source: BMC Microbiol. 2019 Jul 8;19:155. doi: 10.1186/s12866-019-1528-1 (PMC6615096; doi:10.1186/s12866-019-1528-1)
Supplement: Supplementary file 1 — Table S1. Actual values of process variables in 1000 mL of fermentation medium. (DOCX 13 kb) [file 12866_2019_1528_MOESM1_ESM.docx]

**Table S1** Actual values of process variables in 1000 mL of culture medium

| Process variables (g/L) | Low level (-1) | High level (+1) |
| --- | --- | --- |
| Starch (X1) | 15 | 25 |
| KNO3 (X2) | 0.5 | 1.5 |
| NaCl (X3) | 0.4 | 0.6 |
| FeSO4 (X4) | 0.005 | 0.015 |
| MgSO4 (X5) | 0.4 | 0.6 |
| K2HPO4 (X6) | 0.4 | 0.6 |
| Tryptophan (X7) | 1 | 3 |
| Incubation time (Day) (X8) | 5 | 7 |
