# Supplementary material for: Indole-3-acetic acid production by Streptomyces fradiae NKZ-259 and its formulation to enhance plant growth
Source: BMC Microbiol. 2019 Jul 8;19:155. doi: 10.1186/s12866-019-1528-1 (PMC6615096; doi:10.1186/s12866-019-1528-1)
Supplement: Supplementary file 2 — Table S2. The Plackett-Burman experimental design. (DOC 37 kb) [file 12866_2019_1528_MOESM2_ESM.doc]

**Table S2** The Plackett-Burman experimental design

| Exp No. | X1 | X2 | X3 | X4 | X5 | X6 | X7 | X8 | IAA (μg/mL) |
| --- | --- | --- | --- | --- | --- | --- | --- | --- | --- |
| 1 | -1 | -1 | -1 | +1 | +1 | +1 | -1 | +1 | 30.814 |
| 2 | -1 | -1 | -1 | -1 | -1 | -1 | -1 | -1 | 28.115 |
| 3 | -1 | -1 | +1 | +1 | +1 | -1 | +1 | +1 | 30.77 |
| 4 | +1 | +1 | +1 | -1 | +1 | +1 | -1 | +1 | 32.647 |
| 5 | +1 | -1 | -1 | -1 | +1 | +1 | +1 | -1 | 60.261 |
| 6 | +1 | +1 | -1 | +1 | -1 | -1 | -1 | +1 | 60.261 |
| 7 | +1 | -1 | +1 | +1 | -1 | +1 | -1 | -1 | 31.204 |
| 8 | +1 | -1 | +1 | -1 | -1 | -1 | +1 | +1 | 35.991 |
| 9 | -1 | +1 | +1 | +1 | -1 | +1 | +1 | -1 | 45.412 |
| 10 | +1 | +1 | -1 | +1 | +1 | -1 | +1 | -1 | 50.235 |
| 11 | -1 | +1 | -1 | -1 | -1 | +1 | +1 | +1 | 52.058 |
| 12 | -1 | +1 | +1 | -1 | +1 | -1 | -1 | -1 | 27.186 |
