# Supplementary material for: Indole-3-acetic acid production by Streptomyces fradiae NKZ-259 and its formulation to enhance plant growth
Source: BMC Microbiol. 2019 Jul 8;19:155. doi: 10.1186/s12866-019-1528-1 (PMC6615096; doi:10.1186/s12866-019-1528-1)
Supplement: Supplementary file 3 — Table S3. Actual values of process variables in 1000 mL of fermentation medium. (DOC 29 kb) [file 12866_2019_1528_MOESM3_ESM.doc]

**Table S3** Actual values of process variables in 1000 mL of culture medium

| Process variables | Low level (-1) | Central level (0) | High level (+1) |
| --- | --- | --- | --- |
| Starch | 15 | 20 | 25 |
| KNO_3_ | 0.5 | 1 | 1.5 |
| NaCl | 0.4 | 0.5 | 0.6 |
| K_2_HPO_4_ | 0.4 | 0.5 | 0.6 |
| Tryptophan | 1 | 2 | 3 |
| Incubation time (Day) | 5 | 6 | 7 |
