# Supplementary material for: Indole-3-acetic acid production by Streptomyces fradiae NKZ-259 and its formulation to enhance plant growth
Source: BMC Microbiol. 2019 Jul 8;19:155. doi: 10.1186/s12866-019-1528-1 (PMC6615096; doi:10.1186/s12866-019-1528-1)
Supplement: Supplementary file 4 — Table S4. The Box-Behnken experimental design. (DOC 89 kb) [file 12866_2019_1528_MOESM4_ESM.doc]

**Table S4** The Box-Behnken experimental design

| No. | Starch | KNO_3_ | NaCl | K_2_HPO_4_ | Tryptophan | Days | Actual Response of IAA (μg/mL) | Predicted Response |
| --- | --- | --- | --- | --- | --- | --- | --- | --- |
| 1 | 20.00 | 1.00 | 0.50 | 0.50 | 2.00 | 6.00 | 82.022 | 73.890 |
| 2 | 20.00 | 0.50 | 0.50 | 0.50 | 3.00 | 7.00 | 33.841 | 32.796 |
| 3 | 20.00 | 1.50 | 0.50 | 0.50 | 1.00 | 5.00 | 30.739 | 34.059 |
| 4 | 25.00 | 1.50 | 0.50 | 0.60 | 2.00 | 6.00 | 52.071 | 54.609 |
| 5 | 20.00 | 1.50 | 0.40 | 0.50 | 1.00 | 6.00 | 36.947 | 33.162 |
| 6 | 25.00 | 0.50 | 0.50 | 0.40 | 2.00 | 6.00 | 24.022 | 34.544 |
| 7 | 20.00 | 0.50 | 0.60 | 0.50 | 3.00 | 6.00 | 22.288 | 26.577 |
| 8 | 25.00 | 1.00 | 0.50 | 0.60 | 1.00 | 6.00 | 51.575 | 55.047 |
| 9 | 20.00 | 0.50 | 0.40 | 0.50 | 1.00 | 6.00 | 25.547 | 21.311 |
| 10 | 25.00 | 1.00 | 0.60 | 0.50 | 2.00 | 5.00 | 44.951 | 50.613 |
| 11 | 25.00 | 1.00 | 0.60 | 0.50 | 2.00 | 7.00 | 80.403 | 75.446 |
| 12 | 15.00 | 1.50 | 0.50 | 0.40 | 2.00 | 6.00 | 55.752 | 59.980 |
| 13 | 20.00 | 1.50 | 0.50 | 0.50 | 3.00 | 7.00 | 41.124 | 44.646 |
| 14 | 20.00 | 1.00 | 0.40 | 0.40 | 2.00 | 5.00 | 26.124 | 33.395 |
| 15 | 20.00 | 1.00 | 0.40 | 0.60 | 2.00 | 7.00 | 47.752 | 41.443 |
| 16 | 25.00 | 1.00 | 0.40 | 0.50 | 2.00 | 7.00 | 29.875 | 31.179 |
| 17 | 25.00 | 1.00 | 0.50 | 0.40 | 1.00 | 6.00 | 73.717 | 61.832 |
| 18 | 20.00 | 0.50 | 0.50 | 0.50 | 1.00 | 7.00 | 34.553 | 37.041 |
| 19 | 20.00 | 1.00 | 0.60 | 0.60 | 2.00 | 7.00 | 50.885 | 50.953 |
| 20 | 15.00 | 1.50 | 0.50 | 0.60 | 2.00 | 6.00 | 45.385 | 43.194 |
| 21 | 20.00 | 0.50 | 0.40 | 0.50 | 3.00 | 6.00 | 31.544 | 27.066 |
| 22 | 20.00 | 1.50 | 0.50 | 0.50 | 1.00 | 7.00 | 32.69 | 48.891 |
| 23 | 20.00 | 1.50 | 0.40 | 0.50 | 3.00 | 6.00 | 25.004 | 28.916 |
| 24 | 15.00 | 1.00 | 0.50 | 0.40 | 1.00 | 6.00 | 35.075 | 37.207 |
| 25 | 20.00 | 1.00 | 0.40 | 0.40 | 2.00 | 7.00 | 74.42 | 69.869 |
| 26 | 20.00 | 0.50 | 0.50 | 0.50 | 1.00 | 5.00 | 37.836 | 22.208 |
| 27 | 20.00 | 1.00 | 0.50 | 0.50 | 2.00 | 6.00 | 79.637 | 73.890 |
| 28 | 20.00 | 1.50 | 0.60 | 0.50 | 1.00 | 6.00 | 38.429 | 42.672 |
| 29 | 20.00 | 1.00 | 0.60 | 0.60 | 2.00 | 5.00 | 60.199 | 57.760 |
| 30 | 20.00 | 1.50 | 0.60 | 0.50 | 3.00 | 6.00 | 33.097 | 38.427 |
| 31 | 20.00 | 0.50 | 0.60 | 0.50 | 1.00 | 6.00 | 30.788 | 38.130 |
| 32 | 20.00 | 1.00 | 0.50 | 0.50 | 2.00 | 6.00 | 78.000 | 73.890 |
| 33 | 15.00 | 0.50 | 0.50 | 0.40 | 2.00 | 6.00 | 39.403 | 38.130 |
| 34 | 15.00 | 1.00 | 0.60 | 0.50 | 2.00 | 5.00 | 34.062 | 24.442 |
| 35 | 20.00 | 1.00 | 0.50 | 0.50 | 2.00 | 6.00 | 76.226 | 73.890 |
| 36 | 25.00 | 1.00 | 0.40 | 0.50 | 2.00 | 5.00 | 35.327 | 26.346 |
| 37 | 15.00 | 1.00 | 0.60 | 0.50 | 2.00 | 7.00 | 41.354 | 39.275 |
| 38 | 20.00 | 0.50 | 0.50 | 0.50 | 3.00 | 5.00 | 22.788 | 17.962 |
| 39 | 15.00 | 0.50 | 0.50 | 0.60 | 2.00 | 6.00 | 29.203 | 31.344 |
| 40 | 20.00 | 1.00 | 0.50 | 0.40 | 2.00 | 6.00 | 82.363 | 73.890 |
| 41 | 20.00 | 1.00 | 0.60 | 0.40 | 2.00 | 7.00 | 80.602 | 79.38 |
| 42 | 25.00 | 0.50 | 0.50 | 0.60 | 2.00 | 6.00 | 35.752 | 42.758 |
| 43 | 15.00 | 1.00 | 0.40 | 0.50 | 2.00 | 7.00 | 47.947 | 44.521 |
| 44 | 20.00 | 1.00 | 0.50 | 0.50 | 2.00 | 6.00 | 59.150 | 73.890 |
| 45 | 20.00 | 1.50 | 0.50 | 0.50 | 3.00 | 5.00 | 38.535 | 29.813 |
| 46 | 15.00 | 1.00 | 0.50 | 0.60 | 3.00 | 6.00 | 45.788 | 39.387 |
| 47 | 25.00 | 1.50 | 0.50 | 0.40 | 2.00 | 6.00 | 79.996 | 61.395 |
| 48 | 15.00 | 1.00 | 0.50 | 0.40 | 3.00 | 6.00 | 26.673 | 28.172 |
| 49 | 15.00 | 1.00 | 0.40 | 0.50 | 2.00 | 5.00 | 26.341 | 29.687 |
| 50 | 25.00 | 1.00 | 0.50 | 0.40 | 3.00 | 6.00 | 45.097 | 44.377 |
| 51 | 25.00 | 1.00 | 0.50 | 0.60 | 3.00 | 6.00 | 37.956 | 37.591 |
| 52 | 20.00 | 1.00 | 0.40 | 0.60 | 2.00 | 5.00 | 28.323 | 30.250 |
| 53 | 20.00 | 1.00 | 0.60 | 0.40 | 2.00 | 5.00 | 32.221 | 35.906 |
| 54 | 15.00 | 1.00 | 0.50 | 0.60 | 1.00 | 6.00 | 26.783 | 30.421 |

The highest IAA production activity observed was 82.363 μg/mL at run 40 in the above Table.
